# Editorial: Avian senses, immunology, and welfare

**DOI:** 10.3389/fphys.2024.1536343

**Published:** 2024-12-12

**Authors:** F. Nicolas Nazar, Rami A. Dalloul, M. Emilia Fernandez, Eve R. Schneider, Shahram Niknafs, Ryan J. Arsenault, Eugeni Roura

**Affiliations:** ^1^ Microfluidics Cluster UPV/EHU, Analytical Microsystems and Materials for Lab-on-a-Chip Group, Analytical Chemistry Department, University of the Basque Country UPV/EHU, Vitoria-Gasteiz, Spain; ^2^ Department of Poultry Science, University of Georgia, Athens, GA, United States; ^3^ Translational Gerontology Branch, Intramural Research Program, National Institute on Aging, NIH, Baltimore, MD, United States; ^4^ Department of Biology, University of Kentucky, Lexington, KY, United States; ^5^ Centre for Nutrition and Food Sciences, Queensland Alliance for Agriculture and Food Innovation, The University of Queensland, St Lucia, QLD, Australia; ^6^ Food and Feed Safety Research Unit, Southern Plains Agricultural Research Center, U.S. Department of Agriculture - Agricultural Research Service, College Station, TX, United States; ^7^ Centre for Nutrition and Food Sciences, Queensland Alliance for Agriculture and Food Innovation, The University of Queensland, Brisbane, QLD, Australia

**Keywords:** avian immunity, immunometabolism, mucosal immunity, host-pathogen interactions, disease models, cytokines

## Introduction

Ensuring the adaptive success and welfare of birds across diverse ecosystems depends on understanding the intricate relationships between their physiology including how they sense the environment. Insights from avian immunology reveal how birds respond to disease challenges and other stressors, crucial for improving health outcomes especially in light of increasing global demand for poultry products. Birds encounter a myriad of natural and human-driven challenges that disrupt their internal balance, triggering responses across sensory, immune and other physiological systems, all essential for survival and optimal performance. While extensive research has explored these areas independently, a comprehensive understanding requires synthesizing insights across these domains. The overarching aim is to compile and merge findings from avian sensory biology, behavior and immunology to reveal how birds respond to environmental challenges and stressors, enhancing welfare and fostering their survival in diversely challenging ecosystems.

A total of 14 papers are part of this collection: one perspective, two reviews and 11 research papers. The contributions have been grouped under two themes.

1. Avian Senses and Environmental Adaptation

The first theme of this Research Topic highlights the relevance of the senses in the adaptation of wild and domestic birds to the diversity of habitats they are exposed ranging from the wilderness to farms and urban settings. The sense of taste has been largely undermined for many years partially caused by the lack of taste papillae in the avian tongue in contrast to mammals. The review from Niknafs et al. is a comprehensive update on the scientific knowledge to date about the anatomy and physiological function of taste across domestic and wild avian species. The review illustrates how birds have adapted taste sensory systems to dietary profiles in avian herbivores, insectivores, omnivores or strict carnivores amongst others. However, bitter taste in avian species is still an understudied field. A second avian taste manuscript presents the results on the highly developed bitter taste sensitivity in zebra finches (Kumar et al.). In this paper Kumar and co-authors investigate the bitter activation profiles of three zebra finch receptors Tas2r5, -r6, and–r7. In this work the well-developed bitter taste of the zebra finch shows to be particularly tuned to detect cucurbitacin I suggesting a prominent ecological role of this compound for this bird.

The perspective article by Loxdale proposes that the bright, contrasting feather colors of certain European birds may act as warning signals to deter predators, possibly indicating chemical defenses. Such coloration, potentially mimicking toxicity, could allow these birds to avoid predation or gain time to escape, as seen with the avoidance of Eurasian magpies by some predators. Fossesca et al. have presented a review aiming at explaining the scientific evidence of the impact of anthropogenic noise on birds. The review explains effects of noise on the avian auditory processing and discusses species-specific behavioral and physiological responses. Finally, a research article written by Kimball et al. studies vigilance against predators in songbirds. The article uncovers that in breeding season female songbirds maintain full focus on vigilance towards predators.

2. Avian Physiological Resilience and Welfare, and Advances in Avian Immunology and Health.

This second theme is composed by nine original research articles, which explore from the microbiota-gut interaction or the interface of physiology and behavior in the context of avian welfare, to exploring pathological scenarios from an immunological perspective. Aylward et al. indicate that modern broiler chickens face challenges in balancing growth with immune responses, especially around 2 weeks post-hatch. This period is critical as muscle accretion accelerates, leaving them particularly vulnerable to disease challenges due to inadequate resource allocation between growth and immunity. In their paper, Nolin et al. expose that the microbiome is both likely affected by host divergent genetic selection and that it exerts influence on host antibody response by various mechanisms. Li et al. findings indicate that dietary chlorogenic acid (CGA) significantly mitigates the disruption of the ileac barrier, as well as reduces oxidative damage and inflammation caused by high stocking density environment in broiler chickens. As a result, CGA enhances ileac integrity and promotes the presence of beneficial intestinal bacteria.

The fact that an IgY-Fc receptor (FcRY) is a critical IgY receptor that regulates the IgY uptake from the maternal blood circulation into the yolk of avian species, further indicating that the two steps of maternal–newly-hatched IgY transfer are controlled by a single receptor, is described in detail by Okamoto et al. Weston et al. show that natural antibodies are a pool of relatively distinct immunoglobulins, and that antigen specificity may affect interpretation of natural antibody function and comparative immunology.

Three papers deepen in pathological scenarios: Majeed et al. findings suggest that natural Intraepithelial lymphocytes (IEL) with innate and innate-like functions might play a critical role in the host response during subclinical necrotic enteritis, potentially conferring protection against *C. perfringens* infection; Milby-Blackledge et al. data show that mucosal immune responses in broiler chickens after *Salmonella* Typhimurium (ST) infection had increased pro-inflammatory cytokines, chemokines, and colony-stimulating factors indicating influx of immune cells. An unexpected rise in interleukin-10 suggests an immuno-regulatory role. Additionally, elevated vascular endothelial growth factor (VEGF) levels imply potential tissue repair and angiogenesis in ST-infected birds. Last but not least, Mo et al. center on the morphological characterization and cytokine response of chicken bone marrow-derived dendritic cells to infection with highly pathogenic and low pathogenic avian influenza viruses.

Behaviour and welfare interactions are explored by Lundgren and Løvlie reporting promising finding as it shows that higher tryptophan levels can help reduce fearfulness in a non-invasive way, such as through diet rather than injections, making it practical for larger settings. However, given the complexity of the serotonergic system, further research is needed to fully understand its role in influencing behavior in poultry, particularly in production environments.

In conclusion, a wide variety of contributions totalizing 14 items address two main themes: 1) The Avian Senses and Environmental Adaptation and 2) Avian Physiological Resilience and Welfare including Advances in Avian Immunology and Health. The first theme focuses on avian senses and their interaction with the environment. One of the main conclusions is the discovery of the role of the taste system on the adaptation to different dietary regimes and ecosystems. The bitter taste system plays a crucial role in this process by recognizing potential harmful chemicals present in the environment such as in the zebra finch. In addition, the Research Topic showcases how bright feather coloration may serve as a predator deterrent and how anthropogenic noise affects avian behavior. The second theme emphasizes the challenges faced by modern broiler chickens in balancing growth and immune responses, particularly during critical growth phases. Research highlights the role of genetics and diet in influencing gut health and microbiota, along with the importance of natural antibodies and maternal transfer mechanisms. Additionally, insights into pathological scenarios reveal significant immune responses to infections and the interplay between inflammatory processes and protective mechanisms. The exploration of behavior and welfare underscores the potential of dietary interventions to reduce fearfulness in poultry, pointing to the need for further investigation into the underlying biological mechanisms. Overall, this Research Topic of studies contributes valuable knowledge to our understanding of Avian Senses, Immunology, and Welfare.

